# Measuring implementation: development of the implementation process assessment tool (IPAT)

**DOI:** 10.1186/s12913-019-4496-0

**Published:** 2019-10-21

**Authors:** M. Hartveit, E. Hovlid, M. H. A. Nordin, J. Øvretveit, G. R. Bond, E. Biringer, J. Assmus, G. H. Mariniusson, T. Ruud

**Affiliations:** 1 Department of Research and Innovation, Helse Fonna Local Health Authority, Haugesund, Norway; 2 Department of Global Public Health and Primary Care, Faculty of Medicine, University of Bergen, Bergen, Norway; 3 Department of Social Science, Western Norway University of Applied Sciences, Sogndal and Norwegian Board of Health Supervision, Oslo, Norway; 4 Department of Psychiatry, Stavanger University Hospital, Stavanger, Norway; 5 Department of Learning, Informatics, Management and Ethics, Karolinska Institutet, Stockholm, Sweden; 6 IPS Employment Center, Westat, Rivermill Commercial Center, Lebanon, USA; 7 Haukeland University Hospital, Centre for Clinical Research, Bergen, Norway; 8 Division Mental Health Services, Akershus University Hospital, Lørenskog, Norway; 9 Institute of Clinical Medicine, University of Oslo, Oslo, Norway

**Keywords:** Implementation, Readiness, Quality improvement

## Abstract

**Background:**

Implementation science comprises a large set of theories suggesting interacting factors at different organisational levels. Development of literature syntheses and frameworks for implementation have contributed to comprehensive descriptions of implementation. However, corresponding instruments for measuring these comprehensive descriptions are currently lacking. The present study aimed to develop an instrument measuring care providers’ perceptions of an implementation effort, and to explore the instrument’s psychometric properties.

**Methods:**

Based on existing implementation literature, a questionnaire was designed with items on individual and team factors and on stages of change in an implementation process. The instrument was tested in a Norwegian study on implementation of evidence based practices for psychosis. Item analysis, factor structure, and internal consistency at baseline were examined.

**Results:**

The 27-item Implementation Process Assessment Tool (IPAT) revealed large variation between mean score of the items. The total scale scores were widely dispersed across respondents. Internal consistency for the total scale was high (Cronbach’s alpha: .962), and all but one item contributed positively to the construct. The results indicated four underlying constructs: individual stages for behavioural change, individual activities and perceived support, collective readiness and support, and individual perceptions of the intervention.

**Conclusions:**

The IPAT appears to be a feasible instrument for investigating the implementation process from the perspective of those making the change. It can enable examination of the relative importance of factors thought to be essential for implementation outcomes. It may also provide ongoing feedback for leaders tailoring support for teams to improve implementation. However, further research is needed to detect the instrument’s properties later in the implementation process and in different contexts.

**Trial registration:**

ClinicalTrials.gov code NCT03271242 (retrospective registered September 5, 2017).

**Electronic supplementary material:**

The online version of this article (10.1186/s12913-019-4496-0) contains supplementary material, which is available to authorized users.

## Background

There are currently shortcomings in the implementation of evidence-based interventions in most health care services [1, 2]. Many efforts to translate recommended care into everyday practice fail [3]. Well-documented evidence-based interventions, such as psychosocial support for patients suffering from schizophrenia, are found to be offered to less than 10% of patients [3]. One reason is the complexity of implementation. Implementation can be defined as ‘the constellation of processes intended to get an intervention into use within an organization’ [4]. It comprises interactive mechanisms and processes for preparing, conducting and maintaining change at both individual and collective levels of the organisation [4, 5]. To improve our understanding of these, to a large degree unknown processes, the field of implementation science suggests a variety of theories and constructs [4–8]. These are helpful for planning, conducting and evaluating implementation efforts and for implementation research [9]. However, the many theories are partially overlapping, none of which capture the full scope of the field [4]. Literature syntheses and frameworks are developed to provide comprehensive descriptions of implementation [4, 6–8]. These are useful for defining factors to measure across theories in order to investigating implementation, as in the case of the present study [9].

### The consolidated framework for implementation research (CFIR)

The Consolidated Framework for Implementation Research (CFIR) was developed to ‘support the exploration of essential factors that may be encountered during implementation’ [4]. It suggests five major domains of implementation; intervention characteristics, outer setting, inner setting, characteristics of individuals, and process [4]. Intervention characteristics is related to aspects of ‘the new and better way of working’, such as gains and costs and whether the intervention can be easily implemented into existing practice [4]. Outer setting includes pressure from various stakeholders that represent valid perspectives, such as patient organisations, professional associations and networks, and politicians [4, 10]. Inner setting regards structural characteristics, networks and communications, culture, the implementation climate, and readiness for implementation [4]. The theories constituting the fourth CFIR domain, characteristics of individuals, focus on individual knowledge and beliefs about the intervention, the individual’s confidence in being able to achieve implementation and commitment to the organisation, among others [4]. The fifth domain, process, includes planning, engaging, executing, and reflecting and evaluating. It is highly inspired by Deming’s plan–do–study–act model and emphasise the stepwise progression of an implementation effort [11]. Even though the CFIR divides implementation theories into five major domains, the authors highlight the dynamic interplay between factors included in each of the domains [12].

### Individual and collective readiness

The CFIR conceptualises implementation as a set of social processes taking place within a social setting [4]. Implementation is an interactive process where care providers constantly evaluate the pros and cons for supporting the implementation initiative [13]. Readiness for change reflects ‘the extent to which the organisation and its members are inclined to accept, embrace and adopt a particular plan to purposefully alter the status quo’ [14]. The concept regards individuals’ perceptions of the situation and cannot be measured by external observation. The cognitive and affective evaluation by care providers takes place at both an individual and a collective level [15]. Collective readiness regards the team’s shared commitments to and capability of change, and is not merely the mean of individual readiness [16]. The individual and collective levels are expected to have different antecedents and outcomes, and should be measured separately [15].

### The stages of change

The CFIR include theories recognising the process of emotional and cognitive preparation to change [4]. Stages-of-change theories explain implementation as a stepwise progression that care providers must complete, going from unawareness to maintenance of the new way of working [13, 17, 18]. Grol and Wensing suggest five stages for care providers and teams [19, 20]. In the first phase, orientation, care providers take interest and start to involve. The second phase, insight, also include a deeper insight into own routines and an assessment of pros and cons for the particular change. In the third stage, the acceptance stage, a decision to support and intention to change is developed. In the fourth stage the care providers make the change and seek confirmation of the expected gains. Finally, the care providers ensure maintenance by integrating the new way of working into daily practice, according to Grol and Wensing [19]. The progression of these stages among care providers can predict implementation, i.e. stagnation or negative response to any of the stages is likely to indicate high risk of failing.

Quantification is essential for understanding and predicting implementation. However, exploration of the implementation process has been limited by several issues. First, the fragmentation of the theoretical grounding for implementation implies a large set of instruments, each capturing only part of the complexity of implementation. We lack instruments that are able to reveal the interactions between the factors involved. Comprehensive frameworks, such as the CFIR [4], have been used to guide the exploration of implementation processes, but have still not been measured [12, 21] or only partly measured [22]. Second, the existing instruments have been criticised for insufficient exploration of psychometric properties [23, 24]. Finally, most existing instruments were developed to measure attitudes and motivation to implement prior to actual change. Examples are instruments measuring attitude towards evidence-based practice or patient safety issues [25–27]. Research that evaluates the relative contribution of implementation factors across stages in the implementation process is limited [5]. Measuring only before implementation will not improve our understanding of why many efforts start off well but subsequently fail somewhere in the process. The Stages of Implementation Completion (SIC) provides a method for examining the completation of stages expected to constitute the implementation process [28]. Although this measurement can reveal important information regarding the stage at which an effort failed and predict discontinuity, it cannot reveal why stages are not completed. A process-based approach to investigate how individuals perceive and react to influence from the environment during the stages of change is recommended [13].

The present study aimed to develop an instrument to measure care providers’ perceptions of factors seen as essential to accomplish implementation in health care. The instrument should be based on a consolidated description of implementation theories, enable exploration of both the individual and collective level of readiness, and reveal indications for progression of stages of change. The study also aimed to explore the instrument’s psychometric properties at baseline.

## Methods

### Context

The context for testing the Implementation Process Assessment Tool (IPAT) was a cluster randomized study of implementation of four evidence based practices for treatment of psychoses in six Norwegian health trusts (Clinical trial: NCT03271242) [29]. Community mental health centres and hospital units participated in the study. Each of the units received implementation support for one of four selected evidence-based practices (antipsychotic medication, physical health care, family psychoeducation, and illness management and recovery (IMR). Antipsychotic medication and physical health care constituted bundles of recommended care in the Norwegian guideline for treatment of persons with psychoses. For practical reasons they are also defined as ‘practices’ in the following text. The IPAT was developed as a part of this study to explore the implementation process.

### Construct and item development

The development process follows the steps of instrument development described by Prince and colleagues, including the definition of the construct, review of the construct definition, item drafting, item review, and alpha testing [30].

**Fig. 1:**
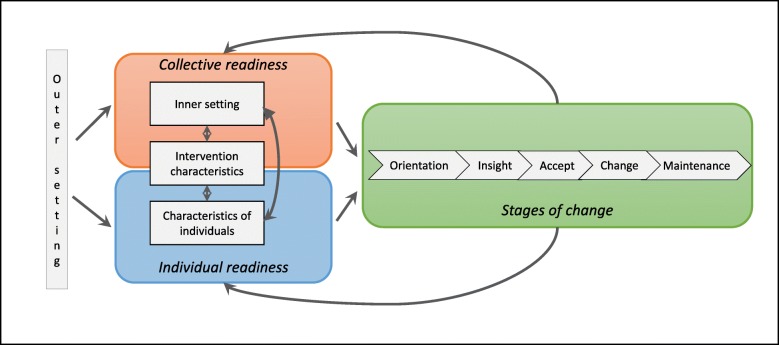
Illustration of the theoretical grounding for the Implementation Process Assessment Tool (IPAT). Factors of the major CFIR domains Outer setting, Inner setting, Intervention characteristics and Characteristics of individuals [4] are constantly being interpreted by care providers, implying readiness at two levels; the individual and the collective. The degree of readiness and the progression of stages of change are expected to interact

- Step 1. Definition of constructs: An expert panel of researchers in implementation science (the authors of this article) compiled the empirical and theoretical literature that was deemed relevant to the aims of the current study. Based on this literature, which was briefly outlined above, we elaborated a model (Fig. 1) to describe the construct of implementation.
- Step 2 and 3. Review of the construct definitions and item drafting: A large set of relevant items to represent essential factors for implementation as defined in the model was developed. We used four criteria for defining items: a) the items should represent the main areas of implementation as described in our model, b) the number of items representing each area should be balanced (i.e. equal weighting between these main areas), c) for feasibility reasons the number of items should be limited implying exclusion of items covered by already suggested items, and d) the items should be defined as social constructs. Existing instruments were consulted for definition of items.
- Step 4. Item review. During a stepwise consensus process [30], the group eliminated or redefined items that were considered to be less appropriate in accordance with the selection criteria. Quality improvement facilitators from mental health care within each of the six participating local health authorities (*N* = 15) assessed the items’ usefulness and sought to detect whether some items were difficult to understand or to answer. The electronic version of the instrument was then tested by care providers at two wards with ongoing local quality improvement projects (one forensic mental ward and one medical ward). These wards were selected for convenience, but also to provide an indication of the face validity within other specialised healthcare contexts. The care providers were asked to give feedback on the content, but also the technical solution, for exploration of the face validity and the usability of the software (Questback) we used.
- Step 5. Testing of the questionnaire and items: Finally, we conducted a full-scale test among a sample of employees in the implementation study, as described in the following.

### Exploration of psychometric properties

#### Sample

Health professionals from 42 clinical units participated in the testing of IPAT. At each unit, the manager was asked to select up to 15 clinicians giving treatment of patients with psychosis. These clinicians received an invitation by e-mail with a link to complete the IPAT for the practice which the unit was to receive implementation support. Non-responders received a reminder 1 month later. At the time of scoring, none of the participants had received systematic implementation support.

#### Analyses

We explored elements of validity using inspection of variance and floor/ceiling effects, exploratory factor analysis, and internal consistency.

Item analysis with regard to variance and floor/ceiling effects were explored descriptively. We inspected variation in scores between items and responders by the means of mean and confidence interval. To explore potential underlying constructs, we conducted exploratory factor analysis. First, an exploratory component analysis was conducted to detect the number of factors, based on a visual inspection of the scree plot [31]. The Eigenvalue was assessed in accordance with the rule of thumb that it should exceed 1 [31]. Second, principal axis factoring with promax rotation was conducted. Oblique rotation was chosen because we considered the variables to be correlated [32]. Items were assigned to the factor upon which they loaded the most. Internal consistency was examined using Cronbach’s alpha for the total scale and each of the factors. For all analysis, we combined the respond to all the four practices to be implemented. Statistical analyses were performed using the Statistical Package for Social Sciences (SPSS) for Windows version 24.0.

## Results

### Construct and item development

The research group defined a model including three main areas of the construct implementation: collective readiness, individual readiness, and stages of change. (See Fig. 1). We understand implementation as a process where care providers continuously interpret and assess factors of outer and inner setting and characteristics of the intervention to decide to support or ignore the implementation initiative. The interpretation and assessment is done at two levels. At the individual level the care providers assess how ‘I think about…’ factors and conditions. At the collective level the care providers assess the collective interpretation of the situation, i.e. how ‘we think about…’. The interpretations at both levels and the progression of change, i.e. the stages of change, are seen as interacting constructs.

The model was operationalised to 27 items for inclusion in the IPAT instrument. The items (except item 20) were scored on a Likert scale from 0 (= not agree/not true) to 5 (= agree/correct). Feedback from the implementation facilitators on the items’ usefulness resulted in three suggestions for minor change regarding the Norwegian wording. The test in the two wards did not reveal any needs for improvement. In Table 1, the IPAT items are presented and described in relation to the theoretical model we developed (Fig. 1), the five major domains in CFIR and short descriptions with reference to more detailed literature.

**Table 1:** Characteristics of the included respondents (*N* = 299)

Characteristics of the included respondents (*N =* 299)	
Gender	
Male	74 (25%)
Female	223 (75%)
Age	
≤ 30 years	23 (8%)
31–40 years	65 (22%)
41–50 years	81 (27%)
≥ 50 years	115 (38%)
Profession	
Psychologist	33 (11%)
Physician	23 (8%)
Nurse	167 (56%)
Social worker	21 (7%)
Other	55 (18%)
Mental health specialist	
Yes	190
No	109

### Sample

In total, 591 health professionals were invited to complete the IPAT, and 375 of these responded (response rate: 63%) at baseline. We stipulated a priori we would exclude any respondent with 4 or more missing items. A total of 76 (20%) respondents were excluded for this reason. Out of these, 45 did not respond to any of the items. Examination of the remaining missing responses revealed equal distribution across items and practices scored, indicating that the missing data was distributed at random. The characteristics of the final sample of health professionals completing IPAT (*N* = 299) is reported in Table 2 and were considered representative for teams in units for treatment of psychosis. The distribution of responses on the four practices were: physical health care (*N* = 113 [38%]), antipsychotic medication (*N* = 54 [18%]), family psychoeducation (*N* = 53 [18%]), or the IMR program (*N* = 79 [26%]).

**Table 2:**
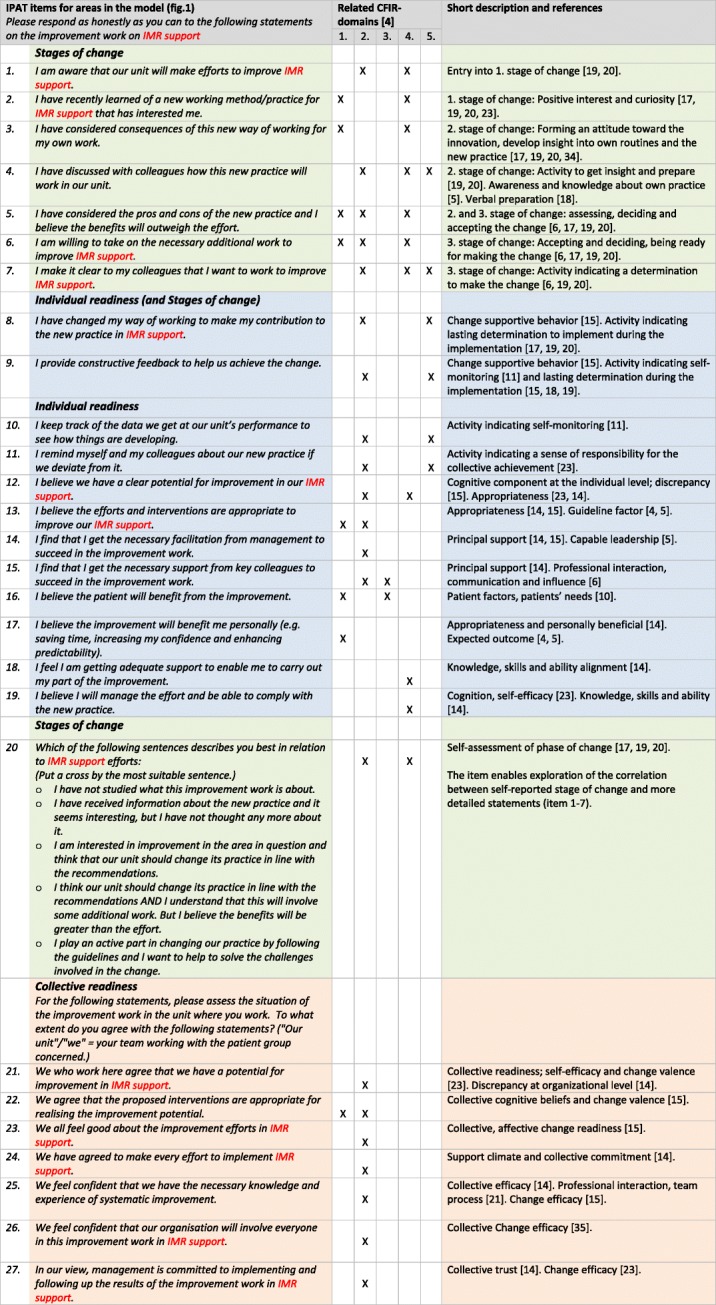
The Implementation Process Assessment Tool (IPAT) items with references. The IPAT items (except item 20) are scored from 0 (= not agree/not true) to 5 (agree/correct). Here we use the questionnaire for an implementation effort on Illness Management and Recovery (IMR), as marked with red. The red text is replaced in questionnaires for other implementation efforts

The areas our model (Fig. 1): Green = Stages of change, blue = Individual readiness, orange = Collective readiness

CFIR-domains: 1 = Intervention characteristics, 2 = Inner setting, 3 = Outer setting, 4 = Characteristics of individuals, 5 = Process

### Exploration of psychometric properties (*N =* 299)

#### Item analysis (variance and floor/ceiling effects)

**Fig. 2:**
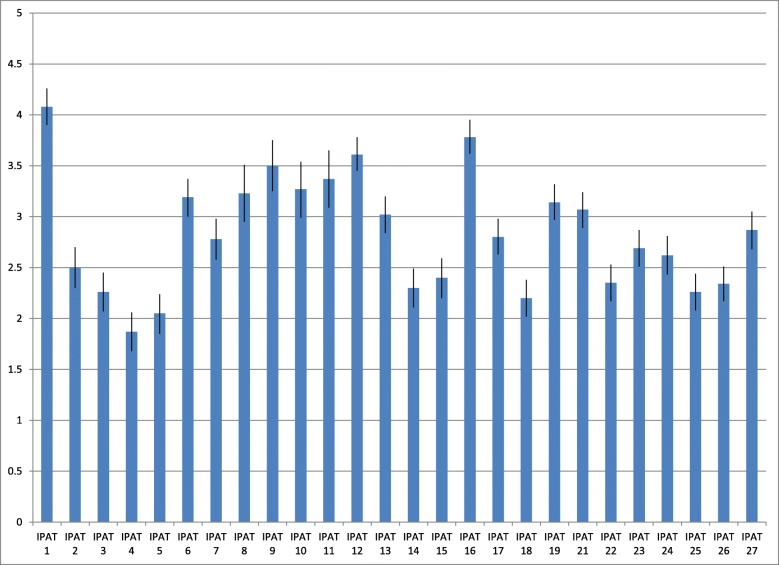
Implementation Process Assessment Tool (IPAT) scores at baseline. Mean score and confidence interval (CI) for each IPAT item at baseline across the four practices (*N* = 299)

The variation we found between respondents for each item and between items indicated sensitivity to inter- and intra-respondent differences. Figure 2 shows the mean and 95% confidence interval (CI) for each item across the four practices. The highest mean score was found for IPAT item 1 *I am aware that our unit will make efforts to improve* [*practice*] *support* (4.08, 95% CI: 3.9–4.26) and the lowest score was found for IPAT item 4 *I have discussed with colleagues how this new practice will work in our unit* (1.87, 95% CI: 1.68–2.06).

Based on the observation that none of the responses were clustered either at the higher or the lower end at the scale, we concluded that ceiling and floor effects are unlikely. For some units, there was substantial variation in how the respondents experienced collective readiness (IPAT items 21–27). For instance, in one unit, three respondents strongly disagreed, one strongly agreed, and the rest gave moderate scored on IPAT item 23, assessing their experience of shared positive feelings regarding the implementation effort in the team.

There was a significant difference, i.e. no overlap between confidence intervals, in the assessments of individual (how ‘I’ assess my situation) and collective assets (how ‘I’ assess the situation in my team). The items allowing this comparison, regarding the potential for improvement, appropriateness of the intervention, and self-efficacy, revealed significantly lower scores for informants’ assessments of the team situation, compared with scores for the corresponding items at the individual level (see Table 3).

**Table 3:** Individual and collective level. Mean and confidence interval (CI) for each of the corresponding items at individual and collective level (*N =* 299)

	Individual level (mean (CI))	Collective level (mean (CI))
Improvement potential		
IPAT 12	3.61 (3.45, 3.78)	
IPAT 21		3.07 (2.89, 3.24)
Appropriateness		
IPAT 13	3.02 (2.84, 3.20)	
IPAT 22		2.35 (2.17, 2.53)
Self-efficacy		
IPAT 19	3.14 (2.97, 3.32)	
IPAT 25		2.26 (2.08, 2.44)

#### Exploratory factor analysis

Visual inspection of the scree plot revealed a drop on the curve from four to five factors (Additional file 1: Figure S1). The eigenvalue of four factors was 1.4, whereas five factors had an eigenvalue at the cut-off of eigenvalue > 1.0. A four factor structure was therefore deemed as the most appropriate structure. The model fit of the principal axis factoring with promax rotation was found to be highly acceptable, with a Kaiser-Meyer-Olkin (KMO) value of .936 and a Bartlett’s Test of Sphericity value of 3793.5 (degrees of freedom: 351, p: .000). The four factors structure explained 69.7% of the variance.

The structure matrix is presented in Table 4, which also shows the factor loadings. The four factors we found are described in Table 5. The first factor included all items defined to measure elements from the stages-of-change literature, except IPAT item 6 *I am willing to take on the necessary additional work to improve* [*practice*] *support*. This item was included in factor 3. However, this item had clear cross-loading for all factors [33]. The third factor included all items regarding collective readiness. The second factor included items regarding activities, as well as perceived support and facilitation from managers and colleagues. The fourth factor included cognitive factors at an individual level. Items regarding the assessment of improvement potential, the appropriateness of the suggested intervention, the probability of compliance, and potential gains for stakeholders loaded on this factor.

**Table 4:** Structure matrix. Structure matrix of principal axis factoring with promax rotation (‘__’ replaces the specification of the practice implemented)

	Factor			
	1	2	3	4
3. I have considered consequences of this new way of working for my own work.	,906	,668	,605	,533
4. I have discussed with colleagues how this new practice will work in our unit.	,892	,629	,544	,487
5. I have considered the pros and cons of the new practice and believe the benefits will outweigh the effort.	,870	,658	,614	,544
2. I have recently learned of a new method/practice for __ support that has interested me.	,840	,637	,626	,579
7. I make it clear to my colleagues that I want to work to improve __ support.	,753	,610	,606	,675
20. Which of the following sentences describes you best in relation to __ support efforts (…).	,662	,497	,479	,560
1. I am aware that our unit will make efforts to improve __ support.	,531		,512	
11. I remind myself and my colleagues about our new practice if we deviate from it.	,629	,873	,542	,476
15. I find that I get the necessary support from key colleagues to succeed in the improvement effort.	,613	,858	,744	,651
8. I have changed my way of working to make my contribution to the new practice in __ support.	,725	,850	,608	,494
9. I provide constructive feedback to help us achieve the change.	,689	,839	,623	,584
18. I feel I am getting adequate support to enable med to carry out my part of the improvement.	,598	,804	,700	,601
14. I find I get necessary facilitation from management to succeed in the improvement work.	,631	,800	,711	,585
10. I keep track of data we get at our unit’s performance to see how things are developing.	,575	,777	,483	,468
24. We have agreed to make every effort to implement __ support.	,644	,592	,866	,528
23. We all feel good about the improvement efforts in __ support.	,651	,576	,827	,535
22. We agree that the proposed interventions are appropriate for realizing the improvement potential.	,756	,666	,812	,633
26. We feel confident that our organization will involve everyone in this improvement work in __ support.		,647	,775	
27. In our view, management is committed to implementing and following up the results of the improvement work in __ support.	,503	,576	,764	
25. We feel confident that we have the necessary knowledge and experience of systematic improvement work to bring about the desired change.		,601	,631	
21. We who work here agree that we have potential for improvement in __ support.	,428		,580	,428
6. I am willing to take on the necessary additional work to improve __ support.	,467	,427	,522	,504
16. I believe the patients will benefit from the improvement.	,539	,468	,483	,845
13. I believe the efforts and the interventions are appropriate to improve our ___ practice.	,565	,612	,616	,812
17. I believe the improvement will benefit me personally (e.g. saving time, increasing my confidence and enhancing predictability).	,486	,471	,445	,776
19. I believe I will manage the effort and be able to comply with the new practice.	,510	,644	,641	,662
12. I believe we have a clear potential for improvement in our __ support.				,424

**Table 5:** Underlying constructs. Underlying constructs in the Implementation Process Assessment Tool (IPAT) with their included items

Suggested underlying constructs	Description	Included IPAT-items
Factor 1: Individual phases for behavioural change	Preparation stages from unawareness to engagement.	IPAT 1–5, 7 and 20.
Factor 2: Individual activities and perceived support	Activities the respondents conduct and their perceived support and facilitation from manager and colleagues.	IPAT 8–11, 14, 15 and 18.
Factor 3: Collective readiness and support	The respondents assessment of “us” and “our” readiness for change and support.	IPAT 21–27 and 6.
Factor 4: Individual perception of the intervention	Improvement potential, ability to comply with the new practice and gains for different stakeholders.	IPAT 12, 13, 16, 17 and 19.

#### Internal consistency

The internal consistency for the total scale was high (Cronbach’s alpha: .962). Further, all items except IPAT item 12 contributed positively to the construct, meaning that excluding one of the items (except item 12) would reduce the internal consistency. After deleting IPAT item 12, Cronbach’s alpha increased by .002. IPAT item 12 asks respondents to respond to the following statement: ‘I believe we have a clear potential for improvement in our [*practice*] support.’ The internal consistency within each of the four factors were slightly lower (Factor 1: .920, Factor 2: .947, Factor 3: .904, and Factor 4: .828).

## Discussion

Based on theories and models included in the CFIR framework, we defined a model of three interacting main areas to be measured when exploring implementation; individual readiness, collective readiness and stages of change. Our operationalisation, the IPAT questionnaire, includes 27 items. Overall, the current exploration of the psychometric properties of this instrument revealed promising results at this early development stage.

A valid operationalisation of the essential factors of implementation should reflect the full scope of the construct in a balanced way [33]. The complexity of the implementation literature, including partially overlapping and interacting constructs, makes operationalization challenging [4, 12]. The 27-item IPAT suggests four underlying constructs: individual stages for behavioural change, individual activities and perceived support, collective readiness and support, and individual perceptions of the intervention. These constructs are in line with the three main areas in our model.

The first construct we found evidence for in these IPAT data is related to the stages in the individual’s change process, as defined by Rogers and by Grol and Wensing [17, 20]. This construct consists of statements to reflect the stages from awareness of a new practice (IPAT item 1 and 2), to actively assessing the practice (IPAT item 3 and 4), deciding (IPAT item 5) and finally, stating support (IPAT item 7). Focusing on the continuous bargain between costs and gains carried out by health professionals during the implementation process is highlighted also within readiness literature [13]. Most comprehensive frameworks for implementation include the concept of stages of change, but have not defined it as a separate major domain [4, 7, 8]. Less is known about how to measure this stepwise progression [4, 34]. The current findings suggest the existence of such a measurable construct.

The third factor, regarding collective aspects, i.e. the shared understanding and commitments in the care unit as seen by the individual care providers, is not defined as a separate major domain in the CFIR. Measuring what Shea termed the ‘supra-individual level’ is recommended for capturing the cooperation and collective effort necessary for achieving organisational changes [35]. The social setting, in which the individuals assess and make their decisions about supporting the implementation effort, is highlighted in most implementation frameworks [36]. Collective readiness (‘how I think about us’) is not equal to the mean individual readiness (‘how I think about me’), and individual readiness is not enough to achieve organisational improvement [35]. The significant difference we found between individual and collective levels for similar factors supports the existence of a separate construct of collective readiness and support. The organisational social context’s impact on individual clinicians’ attitudes toward evidence practices, found by Aarons and colleagues, indicate that the collective attitude and commitment are of importance to understand the development of individual readiness [34].

The second and fourth constructs in the IPAT are individual activities and perceived support, and individual perceptions of the intervention. This deviation of individual readiness into two constructs was not defined originally in our model. However, it is in line with the CFIR which suggests inner setting and intervention characteristics as two major domains [12]. Further, it is in accord with the specification of implementation-level interventions, regarding ways of ensuring the uptake of a new practice, in addition to the intervention-level (the new way of working) [5, 37].

### Strengths and limitations

The IPAT measures how people interpret and feel about a specific implementation effort. One strength of the development of IPAT is that such assessments may reveal the actual impact of implementation factors, in contrast to external observations of potential success factors [5]. It may shed light on causal mechanisms between implementation factors and implementation outcomes. To our knowledge, the IPAT is the first instrument to measure implementation using a process-based approach for exploring readiness factors at both individual and collective levels. By the comprehensiveness of theoretical constructs included, the IPAT may have the potential of revealing interactions between factors and their impact on implementation outcomes which have been missing. The IPAT revealed significant differences in individual and collective level scores. Theoretically, we expect it to be more challenging to achieve collective commitment and capability [14]. However, our instrument is among the first to enable quantitative examination of this difference. Further, we found that respondents within the same organisational unit can experience the collective readiness factors differently. With the IPAT we are able to explore whether large intra-team variance are associated with a greater risk of failure than a generally low score.

Several limitations to what we know about the IPAT’s properties should be considered. Operationalisation implies the translation of many factors into a feasible number of statements. This hinders replicability, and there is a risk that we missed important elements of the ‘true’ construct. Further, this may have obstructed the translation of the results back to theory [37, 38]. We attempted to limit the impact of this shortcoming by explaining how we interpreted the implementation literature into a model including three main areas, then into 27 items. Utilising our experiences in quality improvement and implementation would be expected to strengthen the face validity and feasibility of the instrument [20, 34]. To date, the exploration of the relative importance of the factors included in comprehensive frameworks, such as the CFIR, has consisted mainly of qualitative studies [21]. Even if the IPAT does not capture the full scope of implementation, the proposed instrument constitutes an opportunity for quantitative exploration of how those implementing a new practice perceive and assess the implementation process, which has been missing until now.

There are uncertainties with regard to the existence of four underlying constructs. Not surprisingly, our factor analyses revealed cross-factor loading for several of the 27 items. Several items load on more than one factor. Based on previous reports, interrelations between the factors was expected [4, 5]. In addition, the internal consistency within each of the four factors was slightly lower than the overall internal consistency, indicating that a one-factor structure could be acceptable. However, given the present context, dataset and methods four factors was found appropriate. The recommended four-factor structure offers an insight into implementation that a one-factor structure cannot. We expect the discriminant validity between factors to be limited. Conducting analyses later in the implementation process will reveal to what degree the four-factor structure remains. We recommend interpreting the factor analysis with caution until further examination of factor-structure later in the implementation process is conducted.

The high internal consistency found for the IPAT indicates that the instrument includes items measuring one overall construct, and we found that all of the items contributed to this construct. However, the results should be interpreted with some degree of caution because of the large number of items included. We expect the variance in intra- and inter-item scores to indicate sensitivity to individual interpretations of factors measured (i.e. the true variation in respondents’ interpretations). However, variance in responses may also reflect reliability issues. For instance, items with compound wordings, such as ‘I have considered the pros and cons of the new practice and I believe the benefits will outweigh the effort’ may be confusing to respondents. The wording was chosen to represent a stage of change which imply completion of prior steps (i.e. have considered) before the present step (i.e. concluded about the benefits) [20]. The instrument’s feasibility appears to be high because the vast majority of respondents who began the survey also completed it. Responses with no items’ scored, which constitute most of the missing data, may be due to other factors (e.g. technical issues) rather than low feasibility of the IPAT itself.

### Generalisability

A substantial body of previous research has highlighted the impact of the context on implementation efforts [6, 8, 39]. The IPAT was developed in Norwegian language and tested within specialised mental health care in Norway. However, this instrument is based on internationally acknowledged literature. We believe that the content of the instrument is valid for most similar health services. The sample used in this study does not represent health professionals in general. Rather, the respondents were a purposive sample selected by managers as the essential professionals involved in conducting the planned implementation. The value of measuring the interpretation of professionals who are central during the implementation process is supported by literature on the cardinal role of opinion leaders [16]. Based on the multiple professions and extensive experience represented in the sample, we consider our sample to represent the targeted population. The implementation literature highlight the need for a measure such as the IPAT. Most likely, potential generalisability of findings across practices is therefore high and should be explored.

## Conclusions

Increasing the success rate for implementation efforts in health care is a critical issue. To realise this goal, valid measurement for detecting mechanisms and predicting factors for implementation is needed. Measuring is necessary also for providing ongoing feedback during the implementation process to enable tailored facilitation. In the present study, we developed the IPAT as one step toward responding to this requirement. The current investigation provided promising results for the psychometric properties of the IPAT. We revealed high internal consistency and the observed scores were in accord with the expected pattern. In addition, exploratory factor analyses revealed underlying constructs supported by existing theories. However, further exploration is needed at a later stage to detect the IPAT’s ability to predict implementation outcomes and its psychometric properties later in the implementation process. Over the next 3 years, we will be able to present more information about the IPAT’s properties. The original Norwegian version of the instrument has been tested within specialised mental health care in Norway. The generalisability to other contexts is currently unknown. However, the instrument is based on international acknowledged literature relevant within most health care settings. We believe the IPAT has potential of providing important and valid data across practices. By publishing at this early stage, we welcome cooperation in further development and testing.

### Implications for research and practice

Developing comprehensive, theory-based, and feasible instruments such as the IPAT provides an opportunity to explore how care providers perceive and react to factors thought to be essential for predicting success. For research purposes, the IPAT can quantify which and to what degree implementation factors that are associated with implementation success. Further, we can examine the association between care providers’ interpretations of these factors and implementation outcomes and potential interactions between factors. The significant higher score on individual compared to collective level factors, indicate that special attention should be given to the unit’s shared commitment and capability. For practical use, the IPAT can provide feedback to managers and implementation teams about their effort to facilitate and engage care providers in the implementation. This may enable more systematic implementation support tailored to the care providers’ needs. The IPAT-report may be used by leaders and implementation facilitators to identify which factors should be focused on during the next period of the implementation process to improve progress, reduce waste and increase the probability of success.

## Additional file

Additional file 1: **Figure S1.** The screeplot for the Eigenvalue of the different numbers of factors. The red line indicate the cut-off at Eigenvalue = 1.0. (PPTX 53 kb)

## Data Availability

The datasets analysed during the current study are available from the corresponding author on reasonable request.

## Abbreviations

CFIR: The Consolidated Framework for Implementation Research
CI: Confidence interval
IMR: Illness management and recovery program
IPAT: Implementation Process Assessment Tool

## References

[1] McGlynn EA, Asch SM, Adams J, Keesey J, Hicks J, DeCristofaro A (2003). The quality of health care delivered to adults in the United States. N Engl J Med.

[2] Drake RE, Essock SM (2009). The science-to-service gap in real-world schizophrenia treatment: the 95% problem. Schizophr Bull.

[3] McGovern M, GJ HH, Drake R, Bond GR, Merrens M (2013). Implementing Evidence-Based Practices in Behavioral Health.

[4] Damschroder LJ, Aron DC, Keith RE, Kirsh SR, Alexander JA, Lowery JC. Fostering implementation of health services research findings into practice: a consolidated framework for advancing implementation science. Implement Sci. 2009. 10.1186/1748-5908-4-50. 10.1186/1748-5908-4-50 PMC2736161 19664226

[5] Fixsen DL, Naoom SF, Blase KA, Friedman RM, Wallace F (2005). Implementation research: a synthesis of the literature.

[6] Greenhalgh T, Robert G, Macfarlane F, Bate P, Kyriakidou O (2004). Diffusion of innovations in service organizations: systematic review and recommendations. Milbank Q.

[7] Feldstein AC, Glasgow RE (2008). A practical, robust implementation and sustainability model (PRISM) for integrating research findings into practice. Jt Comm J Qual Patient Saf.

[8] Kaplan Heather C, Provost Lloyd P, Froehle Craig M, Margolis Peter A (2011). The Model for Understanding Success in Quality (MUSIQ): building a theory of context in healthcare quality improvement. BMJ Quality & Safety.

[9] Lynch EA, Mudge A, Knowles S, Kitson AL, Hunter SC, Harvey G (2018). “There is nothing so practical as a good theory”: a pragmatic guide for selecting theoretical approaches for implementation projects. BMC Health Serv Res.

[10] Øvretveit J (1990). Quality health services.

[11] Berwick DM (1996). A primer on leading the improvement of systems. BMJ.

[12] Damschroder LJ, Lowery JC (2013). Evaluation of a large-scale weight management program using the consolidated framework for implementation research (CFIR). Implement Sci.

[13] Stevens GW (2013). Toward a process-based Appoach of conceptualizing change readiness. J Appl Behav Sci.

[14] Holt DT, Vardaman JM (2013). Toward a comprehensive understanding of readiness for change: the case for an expanded conceptualization. J Chang Manag.

[15] Rafferty AE, Jimmieson NL, Armenakis AA (2013). Change readiness a multilevel review. J Manag.

[16] Weiner A (2009). A theory of organizational readiness for change. Implement Sci.

[17] Rogers EM (1995). Diffusion of innovations.

[18] Prochaska JO, DiClemente CC, Norcross JC (1992). In search of how people change: applications to addictive behaviors. Am Psychol.

[19] Grol R, Wensing M, Eccles M, Davis D (2013). Improving patient care: the implementation of change in health care.

[20] Grol R, Wensing M (2004). What drives change? Barriers to and incentives for achieving evidence-based practice. Med J Aust.

[21] Kirk MA, Kelley C, Yankey N, Birken SA, Abadie B, Damschroder L (2015). A systematic review of the use of the consolidated framework for implementation research. Implement Sci.

[22] Walker TJ, Rodriguez SA, Vernon SW, Savas LA, Frost EL, Fernandez ME (2019). Validity and reliability of measures to assess constructs from the inner setting domain of the consolidated framework for implementation research in a pediatric clinic network implementing HPV programs. BMC Health Serv Res.

[23] Weiner Bryan J., Amick Halle, Lee Shoou-Yih Daniel (2008). Review: Conceptualization and Measurement of Organizational Readiness for Change. Medical Care Research and Review.

[24] Gagnon MP, Attieh R, Ghandour EK, Légaré F, Ouimet M, Estabrooks CA (2014). A systematic review of instruments to assess organizational readiness for knowledge translation in health care. PLoS One.

[25] Egeland KM, Ruud T, Ogden T, Lindstrøm JC, Heiervang KS (2016). Psychometric properties of the Norwegian version of the evidence-based practice attitude scale (EBPAS): to measure implementation readiness. Health Res Policy Syst.

[26] Colla JB, Bracken AC, Kinney LM, Weeks WB (2005). Measuring patient safety climate: a review of surveys. Qual Saf Health Care.

[27] Aarons GA (2004). Mental health provider attitudes toward adoption of evidence-based practice: the evidence-based practice attitude scale (EBPAS). Ment Health Serv Res.

[28] Chamberlain P, Brown CH, Saldana L (2011). Observational measure of implementation progress in community based settings: the stages of implementation completion (SIC). Implement Sci.

[29] Ruud T (2017). Implementation of National Guidelines for Treatment of Psychoses.

[30] Prince M, Stewart R, Ford T, Hotopf M (2003). Practical psychiatric epidemiology.

[31] DeVellis RF (2017). Scale development theory and applications.

[32] Yong AG, Pearce S (2013). A beginner’s guide to factor analysis: focusing on exploratory factor analysis. Tutor Quant Methods Psychol.

[33] Bowling A (2009). Research methods in health. Investigating health and health services. 3rd ed.

[34] Aarons GA, Glisson C, Green PD, Hoagwood K, Kelleher KJ, Landsverk JA (2012). The organizational social context of mental health services and clinician attitudes toward evidence-based practice: a United States national study. Implement Sci.

[35] Shea CM, Jacobs SR, Esserman DA, Bruce K, Weiner BJ (2014). Organizational readiness for implementing change: a psychometric assessment of a new measure. Implement Sci.

[36] Nilsen P, Bernhardsson S (2019). Context matters in implementation science: a scoping review of determinant frameworks that describe contextual determinants for implementation outcomes. BMC Health Serv Res.

[37] Grol RPTM, Bosch MC, Hulscher MEJL, Eccles MP, Wensing M (2007). Planning and studying improvement in patient care: the use of theoretical perspectives. Milbank Q.

[38] Foy R, Ovretveit J, Shekelle PG, Pronovost PJ, Taylor SL, Dy S (2011). The role of theory in research to develop and evaluate the implementation of patient safety practices. BMJ Qual Saf.

[39] Harvey G, Jas P, Walshe K, Skelcher C (2015). Analysing organisational context: case studies on the contribution of absorptive capacity theory to understanding inter-organisational variation in performance improvement. BMJ Qual Saf.

