# Supplementary figures and images for: Measuring implementation: development of the implementation process assessment tool (IPAT)
Source: BMC Health Serv Res. 2019 Oct 21;19:721. doi: 10.1186/s12913-019-4496-0 (PMC6805659; doi:10.1186/s12913-019-4496-0)

## Slide 1
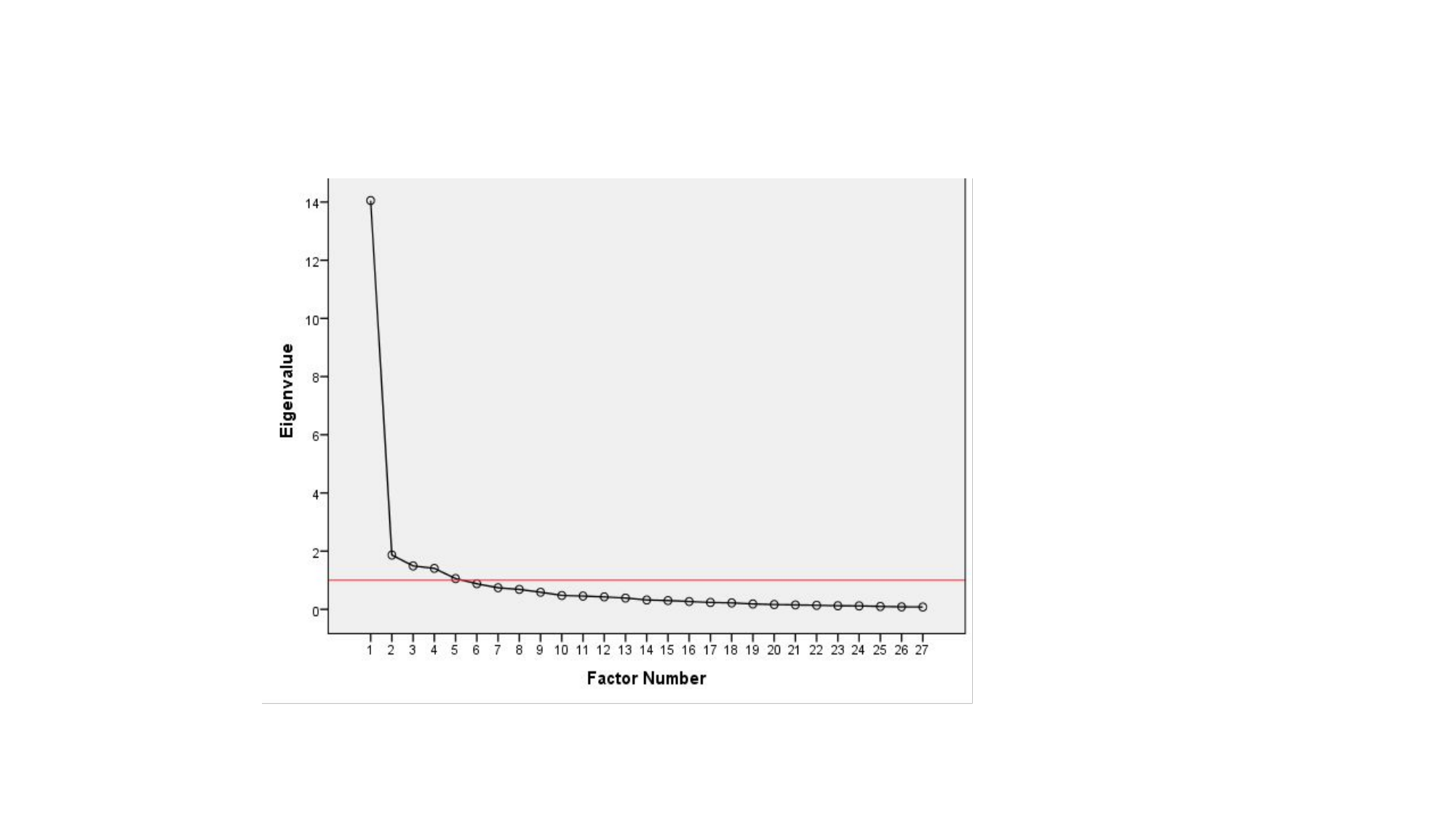

Supplement: Supplementary file 1 — Figure S1. The screeplot for the Eigenvalue of the different numbers of factors. The red line indicate the cut-off at Eigenvalue = 1.0. (PPTX 53 kb) [file 12913_2019_4496_MOESM1_ESM.pptx]
